# Antioxidant Intake and Antitumor Therapy: Toward Nutritional Recommendations for Optimal Results

**DOI:** 10.1155/2016/6719534

**Published:** 2015-11-22

**Authors:** Nuria Mut-Salud, Pablo Juan Álvarez, Jose Manuel Garrido, Esther Carrasco, Antonia Aránega, Fernando Rodríguez-Serrano

**Affiliations:** ^1^Institute of Biopathology and Regenerative Medicine, University of Granada, 18071 Granada, Spain; ^2^Department of Cardiovascular Surgery, Virgen de las Nieves Hospital, 18014 Granada, Spain

## Abstract

The role of the induction of oxidative stress as the mechanism of action of many antitumor drugs is acquiring an increasing interest. In such cases, the antitumor therapy success may be conditioned by the antioxidants present in our own body, which can be synthesized *de novo* (endogenous) or incorporated through the diet and nutritional supplements (exogenous). In this paper, we have reviewed different aspects of antioxidants, including their classification, natural sources, importance in diet, consumption of nutritional supplements, and the impact of antioxidants on health. Moreover, we have focused especially on the study of the interaction between antioxidants and antitumor therapy, considering both radiotherapy and chemotherapy. In this regard, we found that the convenience of administration of antioxidants during cancer treatment still remains a very controversial issue. In general terms, antioxidants could promote or suppress the effectiveness of antitumor treatment and even protect healthy tissues against damage induced by oxidative stress. The effects may depend on many factors discussed in the paper. These factors should be taken into consideration in order to achieve precise nutritional recommendations for patients. The evidence at the moment suggests that the supplementation or restriction of exogenous antioxidants during cancer treatment, as appropriate, could contribute to improving its efficiency.

## 1. Introduction

The first definition of antioxidant was proposed by Halliwell et al. in 1989 as “any substance that, present in low concentrations compared to oxidizable substrates (carbohydrates, lipids, proteins or nucleic acids), significantly delays or inhibits the oxidation of the mentioned substrates” [1]. Later, other definitions of antioxidant were proposed, such as “any substance that prevents, delays or eliminates oxidative damage of a target molecule” [2] or “any substance that can eliminate reactive oxygen species directly or indirectly, acting as a regulator of the antioxidant defense, or inhibiting the production of those species” [3].

**Figure 1:**
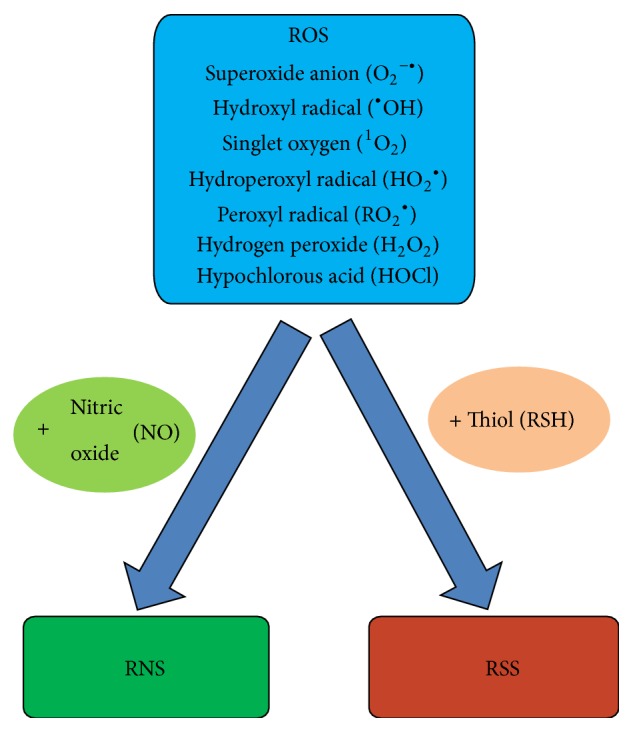
Oxygen reactive species (ROS) and derivatives. ROS includes superoxide anion, hydroxyl radical, singlet oxygen, hydroperoxyl radical, peroxyl radical, hydrogen peroxide, and hypochlorous acid. There are other reactive species which result from the reaction between ROS and nitric oxide (reactive nitrogen species, RNS), or with thiols (reactive sulfur species, RSS) [4–6].

Reactive oxygen species (ROS) are a group of molecules produced by some metabolic processes, due to the action of oxidases in the mitochondria or other cellular compartments. ROS have high reactivity because they possess unpaired electrons that can interact with oxidizable substrates through redox reactions. The main ROS involved in the biological systems are superoxide anion, hydroxyl radical, hydroperoxyl and peroxyl radical, nitric oxide, and other species such as hydrogen peroxide, singlet oxygen, and hypochlorous acid [4, 5]. However, there are other reactive molecules derived from the reaction of ROS with nitric oxide (reactive nitrogen species, RNS) or thiols (reactive sulfur species, RSS) [6] (Figure 1).

The balance between oxidants and antioxidants (redox balance) is essential in maintaining a healthy cellular microenvironment. The generation of oxidative stress is caused by an alteration in the balance between ROS production and the efficiency of the cell antioxidant defense system. Cells and tissues are continuously being exposed to free radicals derived from the metabolism or external factors, such as pollution, microbes, allergens, radiation, cigarette smoke, and pesticides [7]. However, ROS can play a dual role, acting as beneficial or harmful factors [8]. On the one hand, the increase in ROS production generates oxidative stress, a damaging process that can alter cell structures and influences the expression of genes related to accelerated cell aging [9]. Nevertheless, ROS derived from the mitochondrial respiratory chain, at low or moderate concentrations, participate in physiological functions such as in the defense against infections and in the maintenance of redox balance [9, 10].

Cells have several mechanisms to transform and eliminate ROS to avoid their harmful effects. The synergistic action of both antioxidant proteins and enzymes and exogenous antioxidants neutralize free radicals and modulate cell signaling [11]. In fact, numerous studies suggest that antioxidants exert a protective effect against radiation and also prevent the development of many diseases such as cancer, atherosclerosis, stroke, rheumatoid arthritis, neurodegeneration, and diabetes [12, 13].

## 2. The Antioxidant Defense

The natural antioxidant defense is composed of endogenous antioxidants, which are enzymatic and nonenzymatic antioxidants produced by our own body, and exogenous antioxidants, which can be incorporated through the diet or nutritional supplements [14]. Furthermore, there is another group that comprises synthetic antioxidants widely used in the food industry, such as butylated hydroxyanisole (BHA), butylated hydroxytoluene (BHT), propyl gallate (PG), and tert-butylhydroquinone (TBHQ). Several* in vivo* studies carried out in the 80s and the 90s reported some health risks associated with the consumption of synthetic antioxidants [15]. However, this is a controversial issue. A trial conducted in 1993 suggested that the toxic effects produced by BHA and BHT occur only at high doses in long-term treatments [16]. Another study found that the usual intake of BHA and BHT at low doses is not associated with stomach cancer risk [17]. More recently, the European Food Safety Authority (EFSA) studied in depth all the contradictory published data and established that the acceptable daily intakes of 0,25 mg/kg/day for BHA and 1,0 mg/kg/day for BHT are safe for adults and children [18].

**Figure 2:**
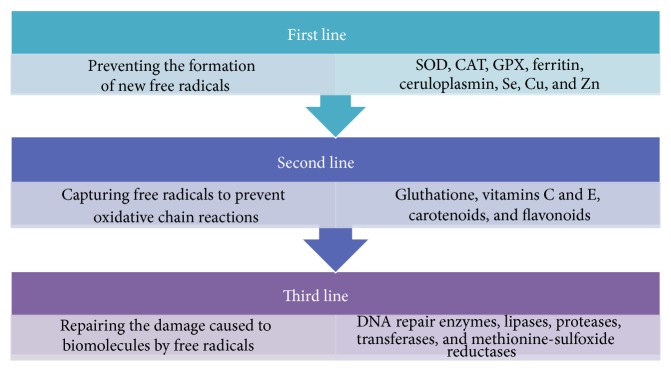
The antioxidant defense. The human antioxidant defense is composed of exogenous and endogenous antioxidants that can be classified into three different lines regarding their mechanism of action. The first line prevents the formation of new free radicals and includes SOD, CAT, GPX, ferritin, ceruloplasmin, Se, Cu, and Zn. The second line captures free radicals to prevent the oxidative chain reactions and includes gluthatione, vitamins C and E, carotenoids, and flavonoids. The third line repairs the damage caused to biomolecules by free radicals and includes DNA repair enzymes, lipases, proteases, transferases, and methionine-sulfoxide reductases [19–21].

Antioxidants can be classified into three lines of defense according to their mechanism of action. The first line includes antioxidants that prevent the formation of new free radicals. It is a very heterogeneous group which includes enzymes such as superoxide dismutase (SOD), catalase (CAT), and glutathione peroxidase (GPX); proteins that bind metals such as ferritin and ceruloplasmin; and minerals such as Se, Cu, and Zn. The second group of antioxidants is responsible for capturing free radicals, and thus they prevent oxidative chain reactions. This group is formed by the glutathione enzyme, albumin, vitamins C and E, carotenoids, and flavonoids. The third line of defense includes antioxidant enzymes that repair the damage caused by free radicals to biomolecules, such as lipases, proteases, DNA repair enzymes, transferases, and methionine-sulfoxide reductases [19–21]. Most exogenous antioxidants are produced by vegetables. Therefore, they are often called phytochemicals, although this is a concept which refers to any chemical compound derived from plants [22] (Figure 2).

## 3. Classification of Exogenous Antioxidants

Exogenous antioxidants constitute a very large and diverse group of molecules in terms of chemical structure and biological properties [23, 24]. Due to the abundance and diversity of members, this group can be divided into three subgroups: polyphenols, vitamins and derivatives, and antioxidant minerals [18].

Polyphenols are the most abundant natural antioxidants. The two main types of polyphenols are flavonoids and phenolic acids. For its part, flavonoids can be classified into several groups: flavonols, flavanones, flavones, catechins, anthocyanins, and isoflavones. Polyphenols are usually secondary metabolites involved in the defense against UV radiation or pathogens [25]. They are found in all plant products such as fruits, vegetables, juices, tea, and wine, and they contribute to their color, taste, smell, and oxidative stability [26]. Numerous epidemiological studies in the late twentieth century have suggested that polyphenols confer some protection against the development of prevalent diseases, including diabetes, infections, cancer, cardiovascular diseases, asthma, and osteoporosis [23, 27, 28].

Within the family of vitamins and derivatives, we want to highlight vitamins C, E, and K and carotenoids. Carotenoids are a group of pigments present in many fruits and vegetables. There are more than 600 types, but only a few of them have demonstrated biological properties, as is the case of *β*-carotene and lycopene. *β*-Carotene is the most studied antioxidants for the prevention of diseases [29]. A product of the hepatic catabolism of *β*-carotene is vitamin A or retinol, which has beneficial effects on the skin, eyes, and internal organs, and that has the ability to combine and neutralize peroxyl radicals before they produce lipid peroxidation [30, 31].

Vitamin C or ascorbic acid is known by its electron-donating ability, thanks to which it prevents the accumulation of oxidizing agents and free radicals. It is especially efficient in eliminating superoxide anion radicals, hydrogen peroxide, hydroxyl, singlet oxygen, and RNS [32, 33]. Vitamin E family includes tocotrienols and tocopherols. They are highly lipophilic molecules that exert an antioxidant action due to their ability to join biological membranes, stabilizing and protecting them against lipid peroxidation [29]. Vitamin K is also lipophilic and it is involved in the blood clotting process. There are two known natural isoforms of vitamin K. K_1_ is present in green plants and is called phylloquinone, while K_2_ types are produced by bacteria of the intestinal flora and are called menaquinones. Although vitamin K is not considered a classic antioxidant, various studies have demonstrated its ability to slow the depletion of glutathione caused by oxidative stress [34].

Within the group of antioxidant minerals, selenium has a special importance because it is a cofactor of antioxidant enzymes such as GPX and thioredoxin reductase, among others [35]. Its role as part of the superoxide dismutase (SOD) and its capacity of inhibiting the NADPG oxidases that catalyze the transformation of oxygen into singlet oxygen radical are also relevant [18]. Similarly, it has been found that zinc can prevent lipid peroxidation and therefore protect cell membranes [36–38].

Apart from the antioxidants mentioned above, in recent years, the importance attributed to melatonin and N-acetylcysteine (NAC) as antioxidants has risen. Melatonin is the main product produced by the pineal gland. It exerts antioxidant activity both directly and indirectly, and it also has anti-inflammatory properties. Melatonin can directly eliminate free radicals such as hydroxyl radical, oxygen singlet, hydrogen peroxide, and peroxynitrite, and indirectly it induces the production of antioxidant enzymes, including GPX, glutathione reductase, Glucose 6P-DH, and SOD. Moreover, unlike classic antioxidants, melatonin does not produce a dose-dependent prooxidant effect, and it is able to cross the blood brain barrier [39]. For its part, NAC has mucolytic properties, it is the precursor of L-cysteine, and it is able to eliminate ROS and restore intracellular glutathione levels. In addition, recent studies indicate that NAC could cross the blood brain barrier, although depending on the dose and method of administration. Both melatonin and NAC also stand out for their low toxicity [40].

## 4. Sources of Exogenous Antioxidants and Diet

Numerous studies have focused on determining the antioxidant content of foods, which conclude that the food with more antioxidant is derived from the plant kingdom (fruits, vegetables, and cereals), while meat and fish are poor in antioxidants. Comparing the group of meat and meat products, with plant foods such as fruits, nuts, cocoa, and berries, the latter are 5- to 33-fold richer in antioxidants than the former [41]. Therefore, diets mainly composed of animal source foods may not provide sufficient antioxidants, which could increase the oxidation of biomolecules and cell damage [42]. Nevertheless, proteins and hydrolysates derived from milk and eggs have shown some antioxidant activity [43].

As stated above, polyphenols are the most abundant group of natural antioxidants. One of them, resveratrol, stands out for its antitumor properties, an aspect that will be discussed later in this paper. This molecule can be synthesized by a large number of plants, in which it seems to protect against different forms of stress such as heat, insects, bacteria, and fungi.

Resveratrol is present in common foods like red grapes and wine, peanuts, and berries [44]. Other important dietary polyphenols are catechins, present in green tea and some fruits [45, 46]; proanthocyanidins, present in many fruits and vegetables, nuts, and seeds [47]; quercetin found in fruits, vegetables, tea, and wine [48, 49]; genistein and daidzein in soy [50]; the phenolic acids in many fruits and vegetables; the hesperetin present in some citrus [51]; the chlorogenic and caffeic acids which abound in coffee [52]; and ferulic acid, found in cereals, citrus fruits, and some vegetables [53]. Cereals, pulses, and nuts also have important polyphenol content [41, 54] (Table 1).

Tea and coffee are very important sources of antioxidants for humans. They are rich in polyphenols and also the two most consumed beverages on the planet after water [54, 55]. Cocoa has a high content of flavanols and procyanidins, and* in vitro* studies have shown that it possesses anti-inflammatory, antiallergic, antiviral, and even antitumor properties [56–58].

Traditional medicinal plants are especially rich in antioxidants, for example, the sap from the trunk of* Croton lechleri* in Peru, known as “sangre de grado” or blood of dragon. This sap has been used for a long time by the Indians of South America to heal wounds, demonstrating antifungal, antiseptic, antiviral, and antihemorrhagic properties. Its main components are proanthocyanidins that have the ability to accelerate the healing of stomach ulcers and to induce apoptosis in some tumor cells [59, 60]. Another interesting plant is* Triphala*, from India, which seems to possess anti-inflammatory, antibacterial, and anticancer properties [61].

Although herbs and spices constitute only a small percentage of the daily food intake, they can be an important source of exogenous antioxidants, especially in cultures where spices are regularly used for cooking [22]. Curcumin is extracted from the plant* Curcuma longa*, and it is commonly used in India. Curcumin has shown anti-inflammatory, antimicrobial, cardioprotective, and neuroprotective properties, among others [62–64]. Recently, it has been found that its mechanism of action involves the expression of antioxidant enzymes such as glutathione transferases, glutathione reductase, and catalase in liver, kidney, and small intestine [64–66]. The content of vitamin C and tocopherols in rosemary (*Rosmarinus officinalis*) [67], sage (*Salvia officinalis*) [68], and cat's claw (*Uncaria tomentosa*) [69] extracts, which are currently used as alternatives sources to synthetic antioxidants in the food industry, has to be highlighted.

Vegetables, fruits, olive oil, and red wine are basic ingredients of the Mediterranean diet. These foods provide a wide variety of antioxidants such as vitamins C and E, polyphenols, and carotenoids [13]. In fact, beneficial effects of the Mediterranean diet, regarding the prevention of cardiovascular diseases and the improvement in cognitive status, have been reported [70, 71].

It is important to note that the antioxidant content of natural products and foods can vary for many reasons, such as the environmental and climatic conditions of growth, storage conditions, and the existence of genetically different varieties [72]. Similarly, the antioxidant content may be substantially modified after processing or cooking. This is the case of berries, which are an important source of flavonoids like tannins, stilbenes, lignans, and phenolic acids. However, during the transformation process of berries into jams and syrup, the content of phenols is reduced up to half of the original amount [72, 73]. However, in other cases, the processing liberates elements included in the food matrix, leading to an increase in the content of certain antioxidants. An example is the tomatoes, since their content in lycopene is available only when they are processed by heat [74, 75].

## 5. Antioxidant Supplementation

Although cells possess a large repertoire of enzymes and antioxidants, sometimes these agents are insufficient to normalize the redox state produced by an intense oxidative stress [76]. In these cases, exogenous antioxidant supplements may be required to restore the cell redox homeostasis [77].

A nutritional or dietary supplement can be defined as any product directed to improve human nutrition and which must contain at least one dietary ingredient. The classic mode of administration of nutritional supplements is orally, in all physical forms, liquid, powder, tablets, capsules, drops, and ampoules. However, in some experimental trials, supplements can be administered by other routes, such as parenteral [78].

The composition of the supplements is very variable. They can contain only vitamins (vitamins C, B and/or multivitamins), only minerals (selenium, zinc, iron, or multimineral), or a combination of vitamins and minerals (multivitamin and multimineral; MVM). Other supplements are mixtures of oils and vitamins or minerals, or plant extracts (ginseng, fiber). Both in Europe and in the United States, the most consumed supplements are MVM, and the most consumed antioxidants are vitamins C, E, D, and A [79, 80]. Additionally, vitamin E is usually added as an antioxidant to preserve different formulations of supplements.

It has been suggested that antioxidant supplementation may protect against oxidative stress associated with the development of certain diseases or that it may reverse the oxidative stress produced during their course. This knowledge has contributed to the fact that the consumption of antioxidant supplements had become an increasingly common practice in the population for the maintenance of physical and mental health [81]. However, the reasons that justify antioxidant supplements consumption vary according to people's age and sex. Usually, older people take them to treat ailments or health problems, while young people consume supplements to achieve higher levels of body energy and to strengthen the immune system. Moreover, women are more likely to use supplements to prevent bone and colon diseases, while men take them to prevent cardiovascular diseases [79, 80].

AHRQ (Agency for Healthcare Research and Quality) in USA conducted a review of all the articles published between 1996 and 2006 related to the preventive effect of MVM supplements on the development of chronic disease [82]. The nutrients considered in the supplements were vitamins D, E, and A, folic acid, calcium, iron, selenium, and *β*-carotene; the diseases were hepatitis, AIDS, rheumatoid arthritis, renal failure, dementia, Parkinson, type II diabetes, cancer, and some ocular diseases (cataracts and macular degeneration), among others. Interestingly, they did not find significant benefits of antioxidant intake for prevention of these diseases and very limited benefit of MVM supplements on primary cancer prevention. The only significant relation found was a reduction in the progression of macular degeneration in smokers who had taken zinc-based supplements.

However, several clinical trials demonstrated that some antioxidant supplements improve the recovery of patients who suffer diseases associated with an excessive production of ROS, for example, premature infants with bronchopulmonary dysplasia (vitamins A, E, recombinant human SOD, Zn, and Se), necrotizing enterocolitis (glutamine, arginine, and human recombinant SOD), periventricular leukomalacia (vitamin E, lactoferrin, and cysteine), or retinopathy (resveratrol, caffeic acid, and epicatechin) [83], and also in cases of idiopathic male infertility (vitamins C, E, coenzyme Q10, glutathione, and selenium) [84]. According to these results, in an experimental assay it was observed that pomegranate juice and resveratrol, orally administered to mice mothers, provide significant protection to their newborn pups against the brain damage caused by hypoxic-ischemic insult. Similarly, supplementation with omega-3 fatty acids could reduce brain damage from rodents, even five weeks after hypoxic-ischemic insult [85, 86].

We have found numerous studies focused specifically on the effect of antioxidant supplementation on cancer and its treatment. These themes will be discussed in the next two sections of this paper.

## 6. Role of Antioxidants in Human Health

Attending to the literature, we believe that antioxidants have impact on health. The questions to be answered are what is the right antioxidant for each particular physiological or pathological condition? And how the antioxidants must be taken, through food or as nutritional supplements? [87].

Many oxidative substances that penetrate into our body through ingestion, inhalation, or skin can be harmful. These substances can generate free radicals that are being accumulated. This accumulation can cause damage and even death due to the biological consequences, whether the antioxidant defense is sufficient or not. Currently, the main causes for reducing the plasma level of antioxidants are smoking and chronic alcoholism [21]. In the skin, for example, there is an antioxidant defense against UV radiation. It is formed by melanin and antioxidant enzymes but also by food antioxidants. This defense prevents swelling, wrinkling, and skin cancer. For that reason, some authors recommend the use of skin protective creams together with antioxidants orally or by topical application, to avoid the damaging effect of sun [88].

**Figure 3:**
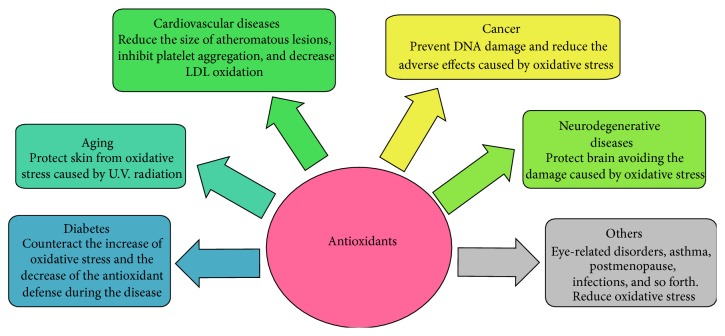
Influence of antioxidants on human health. Antioxidants can influence many aspects of human health such as diabetes, aging, cardiovascular and neurodegenerative diseases, cancer, and other illnesses. Antioxidants produce several beneficial effects, promoting a healthy status, reducing the oxidative stress caused by ROS [89, 90].

The benefit of antioxidant uptake has been demonstrated in the course of some diseases and certain conditions as diabetes, asthma, hemodialysis, thalassemia, rheumatoid arthritis, systemic attack, postmenopause, schizophrenia, depression, and leukemia [89, 90] (Figure 3).

The consumption of polyphenols has been associated with the prevention of the development of atheromatous lesions [91], the reduction of the size of such lesions* in vivo* [92, 93], and the inhibition of platelet aggregation* in vitro* [94] and* in vivo* [95]. In addition, polyphenols seem to reduce the oxidation of LDL, a process that may be responsible for atherosclerosis development. For its part, tea catechins inhibit proliferation and invasiveness of smooth muscle cells in the artery walls of experimental animals. This effect could contribute to reducing the formation of atheromatous lesions. However, this effect has not been fully clarified in humans [91].

Oxidative stress and the damage it causes in the brain are involved in the pathophysiology of highly prevalent neurodegenerative diseases. Several studies suggested that the consumption of foods rich in polyphenols can prevent the development of these diseases [96, 97]. Green tea provides protection against Parkinson [10], and daily consumption of wine has been linked to a lower incidence of dementia and Alzheimer [98]. In fact, it has been shown that dietary polyphenols act against hydrogen peroxide, being more effective than vitamins [99]. Similarly, the consumption of fruit and vegetable juices may also play an important role in delaying the development of neurodegenerative disease [100].

In the area of our interest, that is, cancer, antioxidants are acquiring great importance. It is believed that antioxidants can prevent the development of cancer due to their effects on cell cycle regulation, inflammation, the inhibition of tumor cell proliferation and invasiveness, the induction of apoptosis, and the stimulation of the detoxifying enzyme activity [29, 101]. The antitumor effect of some polyphenols, such as catechins, isoflavones, lignans, flavanones, resveratrol, ellagic acid, quercetin, and curcumin, has been extensively studied. It has been found that these compounds are able to reduce tumor growth through various action mechanisms, in different locations such as mouth, stomach, liver, lung, duodenum, colon, mammary gland, and skin [102–104].

One important antioxidant is resveratrol, since it has demonstrated both* in vivo* and* in vitro* ability to slow down tumor progression in experimental models of lung, skin, breast, and colon cancer, it interferes with the inflammatory mechanisms, and it has antiangiogenic and antimetastatic properties [104–106]. These findings, coupled with the fact that high doses of oral resveratrol seem to be nontoxic, make resveratrol a promising antioxidant for cancer therapy [107].

Regarding the prevention of cancer, there are numerous studies that often provide conflicting conclusions. As an example, a systematic review of lung cancer concluded that there was evidence to recommend supplements of vitamins A, C, and E and selenium, both individually and in combination, to prevent lung cancer. This study also could indicate that the intake of *β*-carotene supplements may be associated with a small increase in the incidence and mortality from cancer in active and passive smokers [108]. This unfavorable aspect of the intake of supplements confirms the results of earlier trials as “The *α*-Tocopherol and *β*-Carotene Trial” (ATBC) and “The Carotene and Retinol Efficacy Trial” (CARET), which also were conducted with smokers. Both studies had to be suspended after observing an increase in the incidence of lung cancer besides an increased mortality due to this cancer [14, 109].

However, some studies have shown reported benefits of consuming antioxidant supplements, such as the trial made by Lappe et al., which showed that supplementation with vitamin D and calcium could reduce the overall risk of cancer in postmenopausal women older than 55 years [110]. In this regard, there is an outstanding study of primary prevention, conducted in large-scale, named NIT (Linxian General Population Nutrition Intervention Trial), which initially involved 29.584 adults of both sexes. This trial evaluated the effect of the intake of supplements of *β*-carotene, selenium, and vitamins E and D, over 10 years in the Chinese town of Linxian. Interestingly, they found a decrease in mortality caused by cancer, especially in stomach cancer. Similarly, it was found that the treatment with supplements tends to be more beneficial in young people. So, the individual's age appears to play a crucial role in the effects obtained [111].

There are some trials that suggest that flavonoids may have a preventive role against colorectal cancer recurrence. One of these studies involved a population of 87 patients who had underwent colon resection or polypectomy and took a supplement composed of a mixture of apigenin and epigallocatechin 3-gallate (EGCG). After 3-4 years of treatment and monitoring by colonoscopy, the results with this long-term treatment appeared to decrease the recurrence of colon cancer in patients with resected colon [112]. Likewise, a similar study found that a high intake of flavonoids was associated with a reduced risk of advanced adenoma recurrence [113]. Among flavonoids, the mentioned EGCG is the major green tea catechin that has been studied more intensively in recent years. Some studies have shown that the intake of this compound can inhibit the disease progression in lung, cervix, breast, stomach, liver, and colon cancer [114]. In addition, numerous clinical trials have been conducted to study the effects of this catechin. One of them involved 8000 patients with stage I or II of breast cancer. Its results revealed that daily consumption of green tea could reduce the recurrence of breast cancer and increase the disease-free survival [114, 115].

The SU.VI.MAX trial is a valuable study that showed controversial results. This study took place in France and included 7876 women aged between 35 and 60 years and 5141 men aged between 45 and 60 years. They were given daily oral supplements in capsules including 6 mg *β*-carotene, 120 mg vitamin C, 30 mg *α*-tocopherol, 100 *μ*g selenium, and 20 mg zinc, individualized or mixed form [140]. The effects of each supplement were evaluated separately and combined. Individual selenium supplementation was associated with some protection against the development of cancer in general in both sexes. This fact has been confirmed by other studies [141]. However, combined therapy consisting of vitamins C and E, *β*-carotene, selenium, and zinc appeared to reduce the incidence of any type of cancer in men but not in women. Researchers attributed this result to the fact that the group of women was younger and less smokers and enjoyed better health than men. Furthermore, previous to the trial, blood tests showed that female samples were higher in vitamin C and *β*-carotene than male samples [140].

Interestingly, supplementation reduced the risk of prostate cancer in 94% of men, while the remaining 6%, who had a higher level of prostate-specific antigen (PSA) in serum, showed an increased risk of developing the disease. It is believed that this beneficial effect would be provided by selenium. This antioxidant mineral may be effective in healthy people or in early stages of the disease, but not in later stages, as in the case of prostate cancer associated with elevated levels of PSA [14, 142]. The intake of selenium as a supplement has shown no effect on the incidence of prostate cancer in patients at high risk for the disease, either with elevated PSA levels or under suspicion of cancer after a digital rectal examination [143]. However, selenium through diet has been associated with a lower risk of pancreatic cancer (up to 20 *μ*gr/day), although this effect seems to disappear if there is an additional intake of MVM supplements that increase the levels of selenium [144].

There is evidence to suggest that the intake of tea and coffee antioxidants, especially vitamin E in form of gamma-tocopherol, would provide some protection against the development of prostate cancer [145, 146]. Similarly, in a clinical trial conducted in Canada, a group of men suffering prostate neoplasia were given daily supplements compound of soy proteins (40 g), vitamin E (800 IU), and selenium (200 mg) for 3 years. It was observed that this supplement appeared to reduce the incidence of prostate cancer [147]. Also, the effect other minerals, such as zinc, could produce on this disease was assessed. Both in* in vitro* and* in vivo* studies, the ability of zinc was found to inhibit the proliferation of prostate tumor cells [148, 149]. Other studies have provided more data about the role of zinc in the course of the disease [150]. Furthermore, the epidemiological study conducted by Leitzmann et al. [151] showed that a high intake of zinc supplementation (>100 mg/day) would increase the risk of prostate cancer, while, according to Ho, the dietary deficiency of this mineral would increase the production of oxidative stress, and, thus, it would increase cell damage both* in vitro* and* in vivo* [152].

A study conducted in Bangladesh, which began in 2006, was to prove the administration of vitamin E and selenium for five years, individually and in combination, to offset the adverse effects of exposure to arsenic suffered by the population The aim was to improve the skin lesions and reduce the incidence of skin cancer caused by arsenic toxicity. However, they found that although the treatment improved the evolution of lesions, there was an increase in mortality and skin dysplasia in the supplemented patients [153]. Similarly, recent* in vivo* studies conducted in mice have shown that the intake of supplements with vitamin E and NAC led to greater progression of lung cancer [154].

## 7. Antitumor Therapy, Oxidative Stress, and Interactions with Antioxidants

Some evidence suggests that cancer cells have a higher level of oxidative stress compared to normal cells. This stress is associated with an increased production of ROS and some changes in the metabolic activity related to oncogenic transformation [155]. Therefore, tumor cells may be more sensitive to drugs that generate big amounts of ROS, or drugs that damage the ROS scavenging capacity of cells, leading these cells to death by apoptosis [156]. Apoptosis is conducted by proteases called caspases, of which there are two main waterfalls, and acts to produce cellular DNA damage and disruption of microtubules [157].

In a multifactorial disease as cancer, an important aspect to consider is the relation between antioxidants and gene expression. Tumor cells show elevated levels of ROS, which may alter prooncogenic signaling pathways that contribute to the malignant phenotype of cells. In this sense, some of the most studied routes are Nrf2 and p53. Nrf2 belongs to an important signaling pathway that controls the expression of genes involved in the neutralization of oxidant agents [158], and the p53 pathway protects the DNA from the oxidation induced by ROS [159, 160]. Many signaling pathways associated with carcinogenesis are related directly or indirectly to ROS metabolism. Thus, these pathways may also be influenced by the presence of antioxidants [161].

Increased ROS during cancer development makes tumor cells become highly dependent on antioxidant agents. For this reason, low concentrations of free radicals due to an excessive administration of antioxidants may promote the proliferation of harmful cells in the neoplastic state, promoting the development of cancer rather than interrupting it [101]. Another aspect to consider is that the intense generation of ROS in tumor cells could damage DNA, promoting the genetic instability and the development of drug resistance. However, it seems interesting to develop new therapeutic strategies to eliminate tumor cells using ROS-mediated mechanisms [155].

Radiation therapy is based on the ability of the ionizing radiation to kill cells. This therapy involves the generation of ROS, including hydroxyl radicals, superoxide anion, and other organic radicals, and also producing lipid peroxidation [126, 162]. In the presence of oxygen, these radicals cause increased formation of other ROS such as peroxides [163]. Therefore, radiation adverse effects would be influenced by these increased radicals, affecting the cellular antioxidant status [164]. In the trial conducted by Bairati et al. with head and neck cancer patients, who were treated with radiotherapy and supplemented with high doses of vitamin C and E, they seemed to improve the adverse effects, but also a loss of effectiveness of the treatment was observed, even an increased mortality in patients who received the treatment with antioxidants [116, 117]. There are several studies that have linked the consumption of these vitamins with improved adverse effects during both chemotherapy and radiotherapy [165–167]. However, other trials showed that the intake of vitamins does not improve the side effects and could even reduce the efficacy of the treatment [168] (Table 2(a)).

Moreover, some studies have reported that curcumin could have synergistic effect with radiotherapy, whether administered separately or in combination [136, 169]. It was observed that, using cell lines of head and neck squamous cell carcinoma (HNSCC), SCC1, SCC-9, A431, and KB, the combination of curcumin and radiation resulted in a greater antitumor effect [124]. The role of curcumin as a radiosensitizer has been supported by the results from other studies, such as the cases of prostate [170], breast [171], colorectal [172], and ovarian tumors [173], among others [62, 174].

Another promising radiosensitizer is EGCG. This catechin has shown synergistic effects with radiation on radioresistant glioblastoma multiforme, multiple myeloma (IM-9), leukemia (K-562), and cancer cervix (HeLa) cells [125]. Moreover, a recent clinical trial showed that EGCG may improve the prognostic of breast cancer patients under radiotherapy [119].

Melatonin is one of the most studied antioxidants in recent years, both in* in vitro* and* in vivo* assays. As it was hypothesized by Vijayalaxmi et al. [175], melatonin may slow the saturation of repair enzymes. This fact would lead to repairing the damage caused by oxidative stress and also would allow the use of higher doses of radiation in the treatment, making melatonin an ideal protective agent during radiotherapy. Although, in most studies, melatonin has been used at very high doses (it is not toxic up to 250 mg/kg), it was found that its administration at low doses in mice, over a period of time (e.g., 0.1 mg/kg/day for 15 days before receiving radiation), appeared to be quite effective, so that the suitable dose of melatonin for humans in radiotherapy treatments is an issue that has to be investigated in more depth [126] (Table 2(b)).

As for chemotherapy, there are numerous agents that induce cell death by oxidative stress either directly, leading to the disruption of redox signaling and ROS scavenging, or indirectly by reducing intracellular levels of antioxidants and deactivating the cellular defense. Numerous articles have reported on many chemotherapeutic agents whose effects involve the induction of oxidative stress. Some of them are new molecules as Meroxest, a synthetic merosesquiterpene derivative of the* trans*-communic acid, plentiful in* Cupressus sempervirens* [176], or Jadomycin, which is synthesized by the bacteria* Streptomyces venezuelae* [177]. Other compounds are part of the current therapeutic repertoire, like oxaliplatin [178], bleomycin [179], gemcitabine [180, 181], cyclophosphamide [182], celecoxib [183], capecitabine [184, 185], bortezomib (a proteasome inhibitor, approved for the treatment of multiple myeloma) [186, 187], and arsenic trioxide (ATO). ATO, which is used in the treatment of acute promyelocytic leukemia (APL), can produce a loss of permeability of the outer mitochondrial membrane and impair the function of the respiratory chain, leading to an increase in superoxide anion [188–191]. However, many of the agents that induce oxidative stress have hardly any studies about the interaction between their antineoplastic activity and antioxidants.

Then, we present the information about various antitumor drugs which have been selected according to their utility, therapeutic efficacy, and involvement in studies that were focused on the evaluation of the interaction with antioxidants during chemotherapy.

Anthracyclines are antitumor antibiotics commonly used in chemotherapy. They have been linked to the generation of oxidative stress and increased ROS levels and could act as mediators of apoptosis by the activation of caspases 3 and 9 [192–194]. Doxorubicin (Adriamycin) is a widely anthracycline used in the treatment of various cancers, including solid breast and prostate tumors. It exerts its antitumor activity by inhibiting topoisomerase II and generating ROS, hereby producing DNA damage and cell death by apoptosis [195, 196]. This increasing of ROS seems to play an important role in the cardiotoxicity caused by doxorubicin [197].

There are* in vitro* studies which have indicated that the administration of antioxidants could counteract the toxicity of this drug in cardiomyoblasts, although other studies have shown different results. For example, vitamin E could exert a cardioprotective effect but only against chronic cardiotoxicity, not against the development of chronic cardiomyopathy [198]. Recently, Wu et al. were able to reduce apoptosis in cardiomyocytes and also the oxidative stress in a model of heart failure in Japanese white rabbits, using intravenous injections of doxorubicin after being treated with NAC [127]. In another experimental trial, it was intended to evaluate the influence of vitamin C on the cytotoxicity caused by antineoplastic agents, such as doxorubicin. As a result, it was observed that when the level of vitamin C was increased, there was a greater resistance to treatment in two cell lines of chronic myelogenous leukemia (K562) and lymphoma (RL). It also occurred in mice with RL cell xenografts. Moreover, after 32 days of treatment, when vitamin C was given to mice 2 hours before being treated with doxorubicin, the tumors became almost four times larger than the tumors of mice treated with just doxorubicin. So, they concluded that vitamin C seemed to interfere with the cytotoxic effect of doxorubicin [128].

In other cases, antioxidant supplements have shown positive effects, without affecting the effectiveness of treatment. The combination of 5-fluorouracil, doxorubicin, and cyclophosphamide (FAC) appears to involve a decrease in antioxidant levels, as a result of the lipid peroxidation produced in the cell membrane [199]. In a clinical trial conducted with patients treated with FAC who were in stage II of invasive ductal carcinoma of breast, the aim was to test the effectiveness of* Uncaria tomentosa*. It was observed that the patients who received chemotherapy along with 30 mg/day of the extract of the plant experienced a decrease of the adverse effects from chemotherapy such as neutropenia, without affecting the effectiveness of drugs [120]. Similarly, tannins (a type of polyphenols) administered during the treatment with doxorubicin showed their capacity of lowering the cardiotoxicity caused by the drug, without reducing its antitumor efficacy.

The ability of EGCG as an adjuvant in chemotherapy has also been investigated both* in vitro* and* in vivo* [125]. This catechin exerts synergistic effects with doxorubicin in chemoresistant models of hepatocellular carcinoma (HCC). In addition,* in vivo* studies showed that mice receiving EGCG with doxorubicin experienced a lower growth rate of liver tumors than mice that received only doxorubicin [130]. Similarly, other trials evaluated the combination of EGCG with other drugs such as 5-FU and cisplatin, and their conclusions also suggest the great potential of this catechin as adjuvant in anticancer therapy [200, 201].

A very important topic in antitumor therapy based on doxorubicin is the development of drug resistance. In this regard, the relationship between this resistance and the presence of endogenous antioxidants was recently described. So, McDonald et al. managed to demonstrate the involvement of peroxiredoxins (Prdx) in the doxorubicin resistance of MCF-7 breast tumor cells. Prdx are a family of six proteins expressed in mammals which are thiol-specific antioxidants. This trial showed that MCF-7 had elevated levels of Prdx compared to nontumor cells MCF-10A, and the levels of these proteins in line MCF-7 resistant to doxorubicin were higher. This study also reported that the suppression of the expression of four of these six Prdx led to increasing the apoptotic effect of doxorubicin [129].

Taxanes are anticancer cytotoxics that include paclitaxel, which is a natural antitumor drug used to treat various types of tumors. Numerous studies have indicated that it induces ROS and alters the permeability of the mitochondrial membrane producing H_2_O_2_. A recent study reported a reduction of glutathione levels in blood samples collected from patients treated with Paclitaxel, which implies that there was a decrease of the antioxidant potential of cells [202].* In vitro* studies also point in the same direction. T47D and MDA-MB231 breast tumor cells, treated with scavengers (NAC, catalase, or SOD), were able to maintain their viability. It was discovered how another agent, such as 2-deoxy-D-glucose (a competitive inhibitor of glycolysis), was able to promote a prooxidant effect of paclitaxel [203]. In other trials, it was shown that the administration of resveratrol, during the treatment with paclitaxel, decreased its antineoplastic action against breast tumor cells both* in vitro* and* in vivo* [131].

Docetaxel (Taxotere) is a derivative of paclitaxel that is often used as a first-line drug to treat prostate cancer and other types of tumors. According to some research, its way of inducing cell death would be due to microtubule depolymerisation [204]. It has also been reported that this drug is able to induce oxidative stress by activating caspase 3 [205, 206]. Recently, the prooxidant effect of docetaxel on breast tumor cells (MDA-231 and MCF-7) was demonstrated, which could be enhanced with the addition of C6 ceramide (a cell-permeable-short-chain ceramide), increasing the drug toxicity [207].

Attending to reduce the side effects of this drug, a reduction of oxidative stress in blood levels of mice with breast tumor cells xenografts was found, due to the supplementation of a nitroxide (3-carbamoylpyrroline nitroxyl derivative pirolin) when they were treated with docetaxel and doxorubicin. It also was found that this compound did not interfere with the antitumor activity of these drugs [132].

Cisplatin was the first heavy metal used for treating cancer and it has been widely used to treat solid tumors of lung, ovary, testes, and lymphoma, among others [208, 209]. Its mechanism of action involves the generation of an intense oxidative stress but also causes numerous side effects due to their toxicity [133, 210]. Its mechanism of action is associated with the expression of p53 (tumor suppressor gene), antiapoptotic Bax proteins, p21 protein (cell cycle regulator), and the cleavage of PARP and caspases 3 and 9 [137]. After an extensive review, it has come to our attention that there is a large literature focused on the study of interactions between treatment with cisplatin and antioxidant supplementation, so this fact may be a reflection of the importance of this drug in the treatment of cancer. Here we report some of the most clarifying studies about this drug.

The role of quercetin is remarkable, since it has been reported in several studies that it seemed to act as an adjuvant in the treatment with cisplatin. In a recent study on the treatment with cisplatin in ovarian tumor cells (C13^*∗*^ and SKOV3), it was found that high concentrations of quercetin (40 *μ*M–100 *μ*M) appeared to have a proapoptotic effect, while low concentrations (5 *μ*M–30 *μ*M) seemed to reduce the damage caused by ROS. This reduction of the damage was due to the increase of SOD, and therefore the antineoplastic effect of cisplatin was attenuated. Similarly, the interaction of quercetin with commonly used drugs in the treatment of ovarian cancer (5-FU, taxol, and pirarubicin) was analyzed and the results were alike. Moreover,* in vivo* studies using athymic nude mice with C13^*∗*^ cells xenografts showed that low doses of quercetin could cause inefficiency in the treatment with cisplatin, 5-FU, taxol, or pirarubicin [133].

Other studies have reported that some antioxidants help to slow the progression of tumor cells. It was discovered that tumor cells of colon (COLO-205-GFP) induced in mice that were treated with cisplatin and received high-dose supplements of vitamins A, E and selenium (5 times higher than the standard diet) along with fish oil experienced a significant lower growth compared to the control tumors [134]. However, the mechanism responsible for this effect has not been explained.

An interesting clinical trial evaluated the effect of vitamin supplementation on the quality of life of patients with cervical cancer, at different stages of the disease and undergoing treatment with cisplatin. In this case, chemotherapy was combined with radiation and cisplatin, and parallely patients took vitamins C, E and *β*-carotene. Most of patients, aged between 29 and 73, displayed lower antioxidant levels than recommended (except for vitamin C and zinc) in the pretrial serum analysis. The results showed that women who took supplements during the treatment had less oxidative damage (lower concentration of free carbonyls in serum), their muscle strength was increased, and they showed less fatigue than women who did not take them. It is noteworthy that, in this study, the dose of supplement contained the recommended daily doses, not like other studies in which doses were much higher [118].

Curcumin is another antioxidant that, in addition to its mentioned radiosensitizer potential, has also been investigated in the role of adjuvant therapy with cisplatin. In an* in vitro* assay performed with liver tumor cells HA22T/VGH, it was reported that curcumin enhanced the cytotoxic activity of the drug [135, 211] and so it did against HNSCC tumor cells (CAL27 and UMSCC lines) both* in vitro* and* in vivo* [136].

There have been numerous studies focused on the study of the effects of NAC during the treatment with cisplatin. It has been reported that the administration of NAC can reverse the cytotoxicity and the proapoptotic effects exerted by cisplatin, in human SKOV3 ovarian carcinoma cells, human B.5 LX-1 SCLC, human U87 glioblastoma cells, and rat Rat1 fibroblasts, reaching values of up to 99% reduction in its efficacy. Interestingly, they found that the proapoptotic effect of cisplatin was blocked by NAC if it was administered before or up to 1 hour later than the drug. In case of adding the antioxidant 8 hours after the cisplatin applying, no changes occurred in the proapoptotic effects [137]. Similarly, thanks to other studies* in vivo*, it has been found that NAC can be otoprotective when it is administered up to 4 hours after cisplatin [138]. It was also found that the best route of administration of NAC, in order to improve protection against the renal damage caused by cisplatin, is the intra-arterial (compared to the oral, intravenous, and intraperitoneal routes) [212]. According to these trials, it appears that both the timing and the route of administration of the antioxidant may be important factors to provide some nutritional recommendations associated with cancer treatment. In addition to NAC, other antioxidants have been evaluated to reduce the toxicity of cisplatin, such as lycopene, which has demonstrated its capability of reducing the renal toxicity induced by this drug [139].

The effect of NAC has also been evaluated in patients treated with combinations of chemotherapy agents plus radiotherapy. A clinical trial, in which 40 children with acute lymphoblastic leukemia (ALL) took part, was intended to assess whether the intake of NAC and vitamin E (400 IU/day) orally would counteract the high toxicity from chemotherapy (vincristine, doxorubicin, cytosine arabinoside, cyclophosphamide, and 6-mercaptopurine) and the prophylactic cranial irradiation during the first two months of treatment. After analyzing blood levels of GPx, malondialdehyde (MDA), tumor necrosis factor-*α* (TNF-*α*), and liver enzymes, the results indicated that children who received antioxidants showed lower incidence of toxic hepatitis and less probabilities of requirement of blood and platelet transfusions during treatment [121].

As mentioned earlier, melatonin has a great antioxidant capacity through different mechanisms of action. Therefore, we have referred to its role as adjuvant in chemotherapy to treat various cancers. In a clinical trial conducted by Lissoni, the effect of administration of 20 mg/day of oral melatonin was evaluated in patients with NSCLC treated with cisplatin plus etoposide or cisplatin plus gemcitabine, or gastrointestinal cancer treated with oxaliplatin and 5-FU. In both cases, patients who received melatonin had a higher rate of tumor regression and a greater two-year survival rate [122]. And more recently, Sookprasert et al. revealed the results of the MIRCIT trial, which concluded that advanced NSCLC patients receiving melatonin in combination with chemotherapy did not get better levels of survival, though the side effects were fewer [123]. It would be necessary to continue further studies to clarify the role of melatonin and to be able to compare these two clinical trials. This would allow us to establish the similarities and differences between the advanced and nonadvanced NSCLC patient, their appropriate treatment, and their basal serum levels of antioxidants, in order to compare the results of both studies.

## 8. Conclusions and Future Perspectives

Considering all the results exposed above, we conclude that antioxidant intake seems to influence the effectiveness of antitumor therapy and its adverse effects. However, we believe that at the moment it cannot be possible to give a general recommendation on whether or not to take antioxidants during treatment. This is because the final effect will depend on the type of cancer, the mechanism of action of the drug or drugs used in the treatment, and the type of antioxidants.

More studies are needed to clarify the results of the clinical trials, which sometimes are contradictory to each other. It is necessary to define the most appropriate patient profiles to adopt a nutritional regimen that could contribute to a better result of antitumor therapy. Aspects such as disease stage, treatment resistance, and previous cycles of chemotherapy and/or radiotherapy may be important factors in this regard.

The variable influence of antioxidants on antitumor therapy can be clearly illustrated considering lung cancer patients, for whom the intake of supplements with high doses of *β*-carotene is harmful, especially in smokers, and it has been correlated with a worse prognosis. However, mineral antioxidants seem to produce a beneficial effect on prostate cancer. Thus, the intake of selenium together with vitamin E, at physiologic doses and for a long period of time, appears to decrease the incidence of the disease. Similarly, zinc appears to inhibit the proliferation of prostate tumor cells. Paradoxically, zinc must be ingested through the diet or as a supplement at physiologic doses (below 100 mg/day), because higher doses can produce the opposite effect. Furthermore, the administration of different antioxidants with the same antitumor drug can also produce very diverse effects. Thus, the intake of vitamin C supplements during the doxorubicin treatment has been associated with an acceleration of the malignant process, whereas if some polyphenols are administered, such as tannins, the systemic toxic effects of the drug could be reduced, without interfering with the efficacy of doxorubicin.

Some clinical studies have shown that certain antioxidants could have synergy with some drugs, enhancing its activity. In this regard, NAC, melatonin, and some flavonoids appear to be the most promising antioxidant candidates for cancer therapies. Since the antioxidant environment has been associated with a reduction of the activity of some drugs, it would be very interesting to conduct new trials, in which one of these antioxidants is administered to patients under nutritional restriction of antioxidants. The antioxidant status of cells has been correlated with the resistance against drugs that exert their antitumor effects through the induction of oxidative stress. Therefore, it would be interesting to evaluate the basal antioxidant status of the patients, by determining serum levels of antioxidants. This study could serve in the future to adjust the doses of exogenous antioxidants. The moment of administration of the exogenous antioxidants seems to be another important factor. In fact, and according to the results of the studies reviewed in this paper, it could be possible that dietary recommendations to patients should not be needed to be followed long time after receiving the drug dose. This is due to the fact that the interaction of antioxidant and drugs seems to disappear a few hours after their administration. Similarly, other important topics are the route of administration and the dosage of antioxidants, since they may affect the effectiveness of the treatment.

It is also important to deepen the understanding of the biochemistry of endogenous antioxidants which are responsible for the failure of some treatments, as in the case of Prdx proteins, which reduce the therapeutic efficacy of doxorubicin. This study would allow the discovery and development of specific inhibitors of such proteins and therefore improve the treatments. In fact, there are already specific inhibitors of SOD and other endogenous enzymes, such as DDT (sodium diethyldithiocarbamate) or ellagic acid, which are being studied to be used to enhance the effect of different cytotoxic agents.

Although it is possible to find in the literature numerous articles that discuss the interaction between cancer and antioxidant therapy, there are many drugs for which this kind of studies has not been performed yet. Future research would be helpful to establish concrete recommendations for patients, in order to improve the response to cancer treatment. In our opinion, it is necessary to define specific guidelines for each type of patient, which have to take into account the following: type of cancer; molecular subtype; stage of the disease; therapy, considering the type of drugs, their mechanisms of action, and the cycles; previous treatment; basal antioxidant status; the type of antioxidants that appear to produce a better response with the selected treatment; route of administration; dosage; and the diet of the patient and habits, with the purpose of correcting the antioxidant intake if some restriction or supplementation is necessary. The main problems related to antioxidants clinical trials may be to define homogeneous groups of patients regarding the histopathological and molecular classification and treatment and to control the pool of serum antioxidant that results from the intake of supplements and habits (diet, alcohol, smoking, etc.). Therefore, these clinical trials should be addressed by multidisciplinary teams comprising at least oncologists and nutritionists.

We believe that nutritional recommendations about exogenous antioxidant supplementation or restriction, as appropriate, carried out in parallel to cancer treatment, could contribute to improving its efficiency.

## Figures and Tables

**Table 1:** Classification of biologically relevant exogenous antioxidants and their natural sources [13, 41].

Exogenous antioxidants	Sources
Vitamins and derivatives	
Vitamin C	Berries, citrus fruits, some vegetables (peppers, cabbage), pulses, and some herbs and spices
Vitamin E	Seeds, vegetable oils, peanuts, nuts, and some fruits
Vitamin K	Green leafy vegetables, some herbs and spices
Carotenoids	
*β*-Carotene	Many vegetables (spinach, carrots, pumpkins, and red pepper) and fruits (mango, apricots, and peaches)
Lycopene	Tomatoes, ketchup, and watermelon

Polyphenols	
Flavonoids	
Quercetin	Fruits (apples, citrus), onions, parsley tea, red wine, and green leafy vegetables
Catechins	Green tea, cocoa, and berries
Proanthocyanidins	Many fruits and vegetables, nuts, seeds, cocoa, and some medicinal herbs
Genistein and daidzein	Soy
Hesperetin	Citrus fruits
Resveratrol	Red grapes, red wine, peanuts, and berries
Phenolic acids	
Caffeic and chlorogenic acids	Coffee
Ferulic acid	Cereals, seeds, citrus fruits, and some vegetables

**(a):** 

Clinical evidence			
Treatment	Disease	Results	Reference
High dose of vitamins C and E + radiotherapy	HNSCC	Improve adverse effects but decrease effectiveness of the treatment	[116, 117]

Normal dose of vitamins C, E and *β*-carotene + cisplatin + radiation	Cervical cancer	Decrease oxidative damage, increased muscle strength, and less fatigue	[118]

EGCG + radiotherapy	Breast cancer	Decrease the levels of angiogenic factors and HGF	[119]

Uncaria tomentosa + FAC	Breast cancer	Decrease the adverse effects without interfering with the efficacy of treatment	[120]

NAC and vitamin E + vincristine, doxorubicin, cytosine arabinoside, cyclophosphamide, and 6-mercaptopurine + radiation	ALL	Decrease the incidence of toxic hepatitis Decrease the requirement of blood and platelet transfusions during treatment	[121]

Melatonin + cisplatin plus etoposide or cisplatin plus gemcitabine	NSCLC	Increase the rate of tumor regression and greater two-year survival rate	[122]
Melatonin + oxaliplatin and 5-FU	Gastrointestinal cancer		

Melatonin in combination with chemotherapy	Advanced NSCLC	Decrease the side effects with no better rates of survival	[123]

HNSCC: head and neck squamous cell carcinoma; ALL: acute lymphoblastic leukemia; NSCLC: non-small-cell lung carcinoma.

**(b):** 

Preclinical evidence			
Treatment	Experimental model	Results	Reference
Curcumin + radiotherapy	SCC1, SCC-9, A431, and KB of HNSCC	Increase the antitumor effect of radiation	[124]

EGCG + radiotherapy	Tumor cervical cells (HeLa), multiple myeloma (IM-9), and leukemic (K-562)	Decrease cell proliferation Increase apoptosis and necrosis	[125]

Melatonin + radiotherapy	CD2-F1 mice	Increase the survival of animals	[126]

NAC + doxorubicin	Model of heart failure in Japanese white rabbits	Decrease apoptosis in cardiomyocytes	[127]

Vitamin C + doxorubicin	Cell lines of chronic myelogenous leukemia (K562) and lymphoma (RL)	Increase the resistance to treatment	[128]
	Mice with RL cell xenografts	Larger tumors in mice	

Suppression of Prdx + doxorubicin	MCF-7 human breast tumor cells	Increase the apoptotic effect of the drug	[129]

ECGC + doxorubicin	Colorectal tumor cells (BEL-7404/DOX)	Increase cell death and the sensitivity to the drug	[130]

Resveratrol + paclitaxel	Human breast tumor cells	Decrease the antitumor action of the drug	[131]

Nitroxide + docetaxel or doxorubicin	Mice with breast tumor cells xenografts	Decrease the side effects without interfering with the efficacy of treatment	[132]

Quercetin + cisplatin or 5-FU, taxol, or pirarubicin	Ovarian tumor cells (C13^*∗*^ and SKOV3)	High concentrations of quercetin: proapoptotic effect Low concentrations of quercetin: decrease the damage caused by ROS	[133]

Quercetin at low doses + cisplatin, 5-FU, taxol, or pirarubicin	Athymic nude mice with ovarian tumor cells (C13^*∗*^) xenografts	Inefficiency in the treatment	[133]

High dose of vitamins A, E and selenium + cisplatin	Tumor cells of colon (COLO-205-GFP) induced in mice	Significant lower growth of tumors compared to the control tumors	[134]

Curcumin + cisplatin	Liver tumor cells (HA22T/VGH)	Increase the cytotoxic effect of the drug	[135]
	HNSCC tumor cells (CAL27, UMSCC)		[136]

NAC before or up to 1 hour after the drug + cisplatin	Human ovarian carcinoma cells(SKOV3), human SCLC tumor cells (B.5 LX-1), human glioblastoma cells (U87), and rat Rat1 fibroblasts	Blocks the proapoptotic effect of the drug	[137]

NAC up to 4 hours after drug + cisplatin	Long-Evans rats	Otoprotective without interfering with the efficacy of treatment	[138]

Lycopene + cisplatin	Adult male Sprague-Dawley rats	Decrease the renal toxicity without interfering with the efficacy of treatment	[139]
